# Association between lifestyle factors and headache

**DOI:** 10.1007/s10194-010-0286-0

**Published:** 2011-01-11

**Authors:** Anke C. Winter, Wolfgang Hoffmann, Christa Meisinger, Stefan Evers, Mechtild Vennemann, Volker Pfaffenrath, Konstanze Fendrich, Sebastian E. Baumeister, Tobias Kurth, Klaus Berger

**Affiliations:** 1 Institute of Epidemiology and Social Medicine, University of Münster, Münster, Germany; 2 Division of Preventive Medicine, Department of Medicine, Brigham and Women’s Hospital, Harvard Medical School, 900 Commonwealth Avenue East, Boston, MA 02115 USA; 3 Institute for Community Medicine, University of Greifswald, Greifswald, Germany; 4 Institute of Epidemiology, Helmholtz Zentrum München, German Research Center for Environmental Health, Neuherberg, Germany; 5 Department of Neurology, University of Münster, Münster, Germany; 6 Neurology Practice Leopoldstrasse, Munich, Munich, Germany; 7 INSERM Unit 708-Neuroepidemiology, Paris, France; 8 University Pierre et Marie Curie, Paris, France

**Keywords:** Migraine, Tension-type headache, Alcohol consumption, Body mass index, Physical activity, Smoking

## Abstract

Modification of lifestyle habits is a key preventive strategy for many diseases. The role of lifestyle for the onset of headache in general and for specific headache types, such as migraine and tension-type headache (TTH), has been discussed for many years. Most results, however, were inconsistent and data on the association between lifestyle factors and probable headache forms are completely lacking. We evaluated the cross-sectional association between different lifestyle factors and headache subtypes using data from three different German cohorts. Information was assessed by standardized face-to-face interviews. Lifestyle factors included alcohol consumption, smoking status, physical activity and body mass index. According to the 2004 diagnostic criteria, we distinguished the following headache types: migraine, TTH and their probable forms. Regional variations of lifestyle factors were observed. In the age- and gender-adjusted logistic regression models, none of the lifestyle factors was statistically significant associated with migraine, TTH, and their probable headache forms. In addition, we found no association between headache subtypes and the health index representing the sum of individual lifestyle factors. The lifestyle factors such as alcohol consumption, smoking, physical activity and overweight seem to be unrelated to migraine and TTH prevalence. For a judgement on their role in the onset of new or first attacks of migraine or TTH (incident cases), prospective cohort studies are required.

**Electronic supplementary material:**

The online version of this article (doi:10.1007/s10194-010-0286-0) contains supplementary material, which is available to authorized users.

## Introduction

Primary headache disorders, including migraine and tension-type headache (TTH), affect millions of people worldwide [1]. The burden of headache has consequences for the individual and the society due to a reduced quality of life, absenteeism and lower performance at work, and increased disease-related costs [2]. Although acute and prophylactic treatments are available, many frequent headache sufferers either do not seek treatment or do not respond satisfactory to these treatment strategies [3]. Behaviour-dependent so-called lifestyle factors have been of interest for the onset of migraine and frequent headaches for a long time. Certain trigger factors for migraine and for TTH have been identified in the past [4, 5]. These trigger factors include alcohol consumption and physical activity [6, 7]. If lifestyle factors trigger specific headache attacks, then they might contribute to the high headache prevalence. In case of an existing association, modification of lifestyle habits would be a sufficient, feasible and cost-effective preventive strategy. Several studies have evaluated the role of lifestyle for the onset of headache in general and for specific headache types such as migraine and TTH [8–19]. They report inconsistent findings. Data on the association between lifestyle factors and probable headache forms, introduced into the headache classification in 2004 by the International Headache Society (IHS) [20], are lacking. The aim of our study was to evaluate the association between four common lifestyle factors including alcohol consumption, body mass index (BMI), physical activity and smoking and headache prevalence (migraine, probable migraine, TTH, probable TTH) in three different German studies applying the 2004 diagnostic criteria of the IHS.

## Methods

### Study population

This analysis is part of the German DMKG Headache Study. To evaluate the association between lifestyle factors and headache, we used data from three studies which were conducted in different regions of Germany: the Study of Health in Pomerania (SHIP) in the northeast of Germany, Cooperative Health Research in the Region of Augsburg (KORA) in the south of Germany and the Dortmund Health Study (DHS) in the west of the country. These regions had been selected for the German DMKG Headache Study with the aim to assess prevalences and consequences of headache types in different areas of Germany [21].

SHIP [22] is a population-based study which was designed to assess a broad range of health and quality of life indicators in the north-east region of Germany after the reunification. The baseline examination was conducted from 1997 to 2001 among the general population of West Pomerania aged 20–79 years using a multistage random sampling design. The initial sample comprised 4,310 participants (final response proportion of 68.8%).

The headache question module was implemented in the first follow-up after 5 years on average. Follow-up response was 83.6%, yielding 3,300 participants aged 25–88 years for this analysis.

KORA [23] is a regional research platform and was established in 1996 to continue and expand the Monitoring Trends and Determinants in Cardiovascular Disease (MONICA) [24] project in Augsburg. In 1994/1995, participants were randomly sampled from the city registries of Augsburg and 16 adjacent communities representing a study area of 600,000 inhabitants. Baseline information including sociodemographic variables, risk factors and medical history were assessed by a standardized face-to-face interview. In addition, all participants underwent a standardized medical examination. The response rate of this baseline survey was 73.1%, yielding a total of 4,856 participants in the age range of 25–74 years. After 10 years of follow-up, the cohort was reassessed. The headache question module was implemented in this follow-up (KORA-F3) which had a response rate of 76%, yielding 2,805 eligible participants aged 35–75 years.

For the DHS, a total sample of 3,820 persons aged 25–75 years was randomly selected from the Dortmund city registration office out of a total population of 591,000 and contacted by mail. Of those sampled, 395 persons were excluded because they had moved out of the study area, died or had no sufficient knowledge of the German language. 3,425 persons were eligible and invited to participate in a personal interview at the study centre. If personal participation at the study centre was impossible, a questionnaire with a subset of the otherwise identical questions was mailed to the participants. The overall response rate was 66.9%, yielding 2,291 participants. For the purpose of this analysis, we only used the data of participants attending the personal interview at the study centre (*n* = 1,312) since headache classification according to the IHS criteria required the extended headache information assessed by the personal interview.

All of the three studies were approved by the responsible ethic committee and all participants gave their informed consent prior to inclusion in the study.

### Headache assessment

Information on headache was assessed identically by standardized face-to-face interviews in all three studies. Participants were asked a standardized headache questionnaire including headache frequency, characteristics and severity. This headache questionnaire was based on the diagnostic criteria of the IHS, 2nd edition, for migraine and TTH. Headache fulfilling all required criteria was classified as complete migraine and complete TTH, respectively. If one criterion was missing, headache was defined as probable migraine or probable TTH according to the most recently diagnostic code of the IHS [20]. The standardized headache question module enabled the ascertainment of the 6-month headache prevalence in general and the 6-month prevalence of complete migraine, probable migraine, complete TTH and probable TTH.

### Assessment of lifestyle factors

Trained interviewers collected data on the following lifestyle factors at the time of the interview in each study: smoking status, alcohol consumption, and physical activity. There were differences between the studies in the methods on how two of the four lifestyle factors were assessed. In KORA and the DHS, assessment of alcohol consumption included the drinking behaviour of the last work day and the last weekend using the same questions. In SHIP, the questions regarding alcohol consumption referred to the last 30 days. Average daily alcohol intake (in grams per day) was then calculated using the beverage-specific quantity–frequency measure: number of days with alcohol consumption (beer, wine, and spirits) multiplied by the average daily alcohol consumption over the past week (KORA/DHS) or month (SHIP), respectively. The method used for estimating the mean daily alcohol consumption is an established and validated method implemented in the WHO MONICA project [25]. Gender-specific alcohol consumption was calculated based on the alcohol consumption in gram per day. Moderate alcohol consumption was defined as a daily alcohol consumption of 1–20 g/day for males and 1–10 g/day for females. An alcohol consumption of ≥20 g/day for males and ≥10 g/day for females was considered as increased alcohol consumption. Participants who consumed <1 g alcohol/day were classified as no-drinkers.

For physical activity, the ascertainment differed between the three studies. In the DHS, physical activity in hours per week was assessed by one question. In KORA, physical activity was distinguished in exercise during summer and exercise during winter. Based upon this information, we have averaged the total amount of physical activity in hours per week over the year. In SHIP, the type of physical activity was also assessed. To achieve comparability in these cases, we distinguished three categories representing the amount of physical activity per week: no physical activity, ≤2 h/week, >2 h/week. Since the participants’ interviews were distributed over a period of three full years in SHIP and 1 year in the DHS, at least one full year of data assessment is covered which facilitate comparability to the averaged amount of physical activity in KORA. Smoking status was divided into the following categories: non-smokers, ex-smokers and current smokers. The category current smokers included participants who smoked on a regular and irregular basis. Height and weight were measured in all three studies following the original MONICA protocol for BMI measurement and the BMI was subsequently derived. The following BMI categories were defined: <25, 25.0–29.9 and ≥30 kg/m^2^. Finally, we summarized the four life style factors in one individual health score by adapting a previously proposed health index [26]. This score included the following variables: smoking status, BMI, gender-specific alcohol consumption and physical activity. We calculated the health index by assigning scores of 0–2 to each individual variable category, for which a higher point value indicates a healthier behaviour. The health score could take values within the range of 0–8. Healthiest behaviour was defined as never smoking, consumption of <1 g alcohol/day, a BMI of <25 kg/m^2^ and physical activity of more than 2 h/week. The three data sets provided additional information on baseline characteristics including age, sociodemographic data, and cardiovascular disease risk factors.

### Statistical analysis

To provide comparability between the three data sets, we restricted our analyses to the age groups 35–75 years as previously done [21]. We used logistic regression models to evaluate the association between different lifestyle factors and headache status. Lifestyle factors included BMI (<25, 25–29.9, ≥30 kg/m^2^), smoking status (never, past, current), gender-specific alcohol consumption (never, moderate, increased) and physical activity (never, ≤2, >2 h/week). Participants with no history of the specific headache type and the respective lifestyle factor were chosen as reference group. Logistic regression models were adjusted for age (continuous) and sex. Additional adjustment for other lifestyle factors and cardiovascular disease risk factors did not indicate different associations. We further calculated age- and sex-adjusted logistic regression models with the health score as independent and every headache type as dependent variable. Participants with missing values for either the exposure or outcome variable were excluded from the corresponding analysis.

For all analyses, we used STATA (version 9.0, StataCorp LP, College Station, TX, USA). A two-tailed *p* value of < 0.05 was considered statistically significant.

## Results

A total of 7,417 individuals participated in the three studies; 2,805 in KORA, 3,300 in SHIP and 1,312 in the DHS.

Baseline characteristics of participants according to the three different study regions are summarized in Table 1. Because of different age ranges in the three studies, we additionally present the common age group of 35–75 years old for all study regions and additionally all participants in the DHS and in KORA. To provide comparability among the three studies, further analyses (Tables 2, 3, 4, 5) were restricted to participants in the age range of 35–75 years yielding a total sample size of 1,134 in the DHS and 2,597 in SHIP. Due to missing values on one of the exposure status variables, we had to additionally exclude a total number of 7 participants in the DHS, 213 participants in SHIP and 7 participants in KORA.

**Table 1:** Characteristics of the regional study populations

	DHS (Dortmund)	KORA (Augsburg)	SHIP (Pomerania)
	*n* = 1,134^a^ (*n* = 1,312)^b^	*n* = 2,805^a^	*n* = 2,597^a^ (*n* = 3,300)^c^
Characteristic			
Age (range)	35–75 (25–75)	35–75	35–75 (25–80)
Mean age (years)	55.6 (52.1)	54.6	54.9 (54.5)
Women (%)	52.4 (52.9)	52.2	52.3 (51.9)
School education ≤9 years (%)	54.7 (49.5)	57.6	40.7 (40.5)
Current smoker (%)	24.8 (25.5)	20.3	25.7 (25.7)
Physical activity >2 h/week (%)	31.1 (32.1)	36.3	22.4 (22.2)
Median alcohol intake (g/d)	2.9 (2.9)	6.8	4.9 (4.4)
BMI ≥ 30 kg/m^2^ (%)	28.8 (26.4)	25.9	32.4 (30.4)
Mean systolic blood pressure (mmHg)	143.0 (140.8)	130.0	133.1 (132.5)
Mean diastolic blood pressure (mmHg)	87.9 (86.8)	82.5	82.7 (81.4)
History of hypertension (%)	38.3 (35.2)	44.2	51.6 (50.4)
History of myocardial infarction (%)	4.3 (3.7)	3.1	4.0 (4.3)
History of diabetes (%)	8.7 (7.6)	6.7	10.4 (11.2)
History of stroke (%)	2. 6 (2.2)	1.7	2.7 (3.0)
History of cancer (%)	5.1 (4.6)	5.0	5.4 (5.8)
Any headache during the last 6 months (%)	45.3 (48.6)	54.6	45.0 (45.5)
Migraine (%)	12.7 (13.0)	10.5	9.7 (9.5)
Complete criteria (%)	8.0 (8.5)	7.9	4.4 (4.3)
Probable criteria (%)	4.7 (4.5)	2.6	5.3 (5.2)
Tension-type headache (%)	24.2 (26.8)	35.0	27.4 (27.7)
Complete criteria (%)	15.7 (17.5)	23.6	15.5 (15.8)
Probable criteria (%)	8.5 (9.2)	11.4	11.9 (11.9)

^a^Age group 35–75 years

^b^Age group 25–75 in Dortmund Health Study (DHS)

^c^Age group 25–88 years in the Study of Health in Pomerania (SHIP)

**Table 2:** Age- and gender-adjusted associations between complete migraine prevalence and lifestyle variables, restricted to age groups 35–75 years

Complete migraine	DHS (*n* = 91)	KORA (*n* = 222)	SHIP (*n* = 114)
	OR (95% CI)	OR (95% CI)	OR (95% CI)
Smoking			
Never	1.00	1.00	1.00
Past	1.74 (1.03, 2.94)	0.91 (0.65, 1.28)	1.00 (0.62, 1.59)
Current	0.96 (0.53, 1.73)	1.09 (0.76, 1.57)	0.73 (0.44, 1.19)
Physical activity			
None	1.00	1.00	1.00
≤2 h/week	0.95 (0.55, 1.62)	0.98 (0.67, 1.43)	1.10 (0.67, 1.80)
>2 h/week	0.67 (0.39, 1.15)	1.25 (0.86, 1.81)	1.28 (0.81, 2.02)
Alcohol consumption^a^			
None	1.00	1.00	1.00
Moderate	0.81 (0.48, 1.39)	1.03 (0.74, 1.43)	0.83 (0.52, 1.33)
Increased	0.75 (0.43, 1.31)	0.58 (0.40, 0.84)	0.60 (0.30, 1.20)
Body mass index (kg/m^2^)			
<25	1.00	1.00	1.00
25.0–29.9	0.80 (0.47, 1.37)	1.19 (0.85, 1.66)	1.03 (0.64, 1.64)
≥30.0	1.05 (0.59, 1.88)	0.95 (0.64, 1.41)	1.12 (0.68, 1.83)
Health index^b^	0.93 (0.82, 1.05)	1.02 (0.94, 1.11)	1.05 (0.93, 1.18)

^a^Gender specific alcohol consumption—men: moderate = 1–20 g/day, increased = ≥20 g/day; women: moderate = 1–10 g/day, increased = ≥10 g/day

^b^Sum of the following life style factors: smoking, physical activity, alcohol consumption, BMI

**Table 3:** Age- and gender-adjusted associations between probable migraine prevalence and lifestyle variables, restricted to age groups 35–75 years

Probable migraine	DHS (*n* = 53)	KORA (*n* = 72)	SHIP (*n* = 137)
	OR (95% CI)	OR (95% CI)	OR (95% CI)
Smoking			
Never	1.00	1.00	1.00
Past	1.48 (0.77, 2.82)	1.49 (0.85, 2.61)	0.91 (0.57, 1.47)
Current	0.90 (0.42, 1.93)	1.89 (1.03, 3.46)	1.55 (1.02, 2.35)
Physical activity			
None	1.00	1.00	1.00
≤2 h/week	2.10 (1.07, 4.12)	0.99 (0.55, 1.76)	1.03 (0.66, 1.61)
>2 h/week	1.31 (0.65, 2.66)	0.74 (0.39, 1.38)	0.81 (0.52, 1.27)
Alcohol consumption^a^			
None	1.00	1.00	1.00
Moderate	0.85 (0.44, 1.65)	0.39 (0.21, 0.75)	0.65 (0.42, 1.01)
Increased	0.75 (0.37, 1.52)	0.60 (0.33, 0.99)	0.50 (0.27, 0.94)
Body mass index (kg/m²)			
<25	1.00	1.00	1.00
25.0–29.9	2.10 (1.00, 4.40)	0.90 (0.50, 1.63)	1.39 (0.91, 2.11)
≥30.0	2.52 (1.15, 5.49)	1.26 (0.68, 2.32)	0.94 (0.58, 1.54)
Health index^b^	0.94 (0.80, 1.10)	0.80 (0.70, 0.92)	0.94 (0.85, 1.05)

^a^Gender specific alcohol consumption—males: moderate = 1–20 g/day, increased = ≥20 g/day; females: moderate = 1–10 g/day, increased = ≥10 g/day

^b^Sum of the following life style factors: smoking, physical activity, alcohol consumption, BMI

**Table 4:** Age- and gender-adjusted associations between complete tension-type headache prevalence and lifestyle variables, restricted to age groups 35–75 years

Complete TTH	DHS (*n* = 178)	KORA (*n* = 663)	SHIP (*n* = 401)
	OR (95% CI)	OR (95% CI)	OR (95% CI)
Smoking			
Never	1.00	1.00	1.00
Past	1.05 (0.70, 1.55)	0.86 (0.70, 1.06)	1.34 (1.04, 1.74)
Current	1.25 (0.83, 1.89)	0.81 (0.64, 1.03)	0.91 (0.68, 1.21)
Physical activity			
None	1.00	1.00	1.00
≤2 h/week	1.17 (0.78, 1.73)	1.09 (0.87, 1.37)	1.20 (0.91, 1.59)
>2 h/week	0.91 (0.61, 1.34)	0.93 (0.74, 1.18)	0.91 (0.69, 1.19)
Alcohol consumption^a^			
None	1.00	1.00	1.00
Moderate	0.98 (0.67, 1.45)	1.03 (0.82, 1.29)	1.35 (0.98, 1.86)
Increased	0.89 (0.60, 1.33)	1.00 (0.80, 1.25)	1.18 (0.80, 1.75)
Body mass index (kg/m^2^)			
<25	1.00	1.00	1.00
25.0–29.9	0.64 (0.43, 0.94)	0.96 (0.77, 1.19)	0.96 (0.74, 1.25)
≥30.0	0.74 (0.49, 1.14)	1.07 (0.84, 1.36)	0.83 (0.62, 1.10)
Health index^b^	0.99 (0.91, 1.09)	1.01 (0.96, 1.07)	1.08 (1.01, 1.15)

^a^Gender specific alcohol consumption—males: moderate = 1–20 g/day, increased = ≥20 g/day; females: moderate = 1–10 g/day, increased = ≥10 g/day

^b^Sum of the following life style factors: smoking, physical activity, alcohol consumption, BMI

**Table 5:** Age- and gender-adjusted associations between probable tension-type headache prevalence and lifestyle variables, restricted to age groups 35–75 years

Probable TTH	DHS (*n* = 96)	KORA (*n* = 319)	SHIP (*n* = 308)
	OR (95% CI)	OR (95% CI)	OR (95% CI)
Smoking			
Never	1.00	1.00	1.00
Past	0.99 (0.59, 1.66)	1.14 (0.87, 1.49)	1.05 (0.78, 1.41)
Current	1.29 (0.75, 2.19)	1.04 (0.75, 1.43)	0.94 (0.69, 1.29)
Physical activity			
None	1.00	1.00	1.00
≤2 h/week	1.08 (0.65, 1.79)	1.02 (0.76, 1.37)	0.84 (0.60, 1.17)
>2 h/week	0.78 (0.47, 1.30)	0.81 (0.59, 1.10)	0.93 (0.69, 1.25)
Alcohol consumption^a^			
None	1.00	1.00	1.00
Moderate	0.87 (0.53, 1.44)	1.25 (0.93, 1.68)	0.87 (0.63, 1.19)
Increased	0.86 (0.51, 1.44)	0.92 (0.68, 1.24)	0.64 (0.42, 0.98)
Body mass index (kg/m^2^)			
<25	1.00	1.00	1.00
25.0–29.9	1.03 (0.61, 1.76)	0.76 (0.57, 1.01)	1.43 (1.05, 1.96)
≥30.0	1.23 (0.70, 2.16)	0.90 (0.66, 1.24)	1.50 (1.08, 2.10)
Health index^b^	0.92 (0.82, 1.04)	1.01 (0.94, 1.08)	0.97 (0.90, 1.04)

^a^Gender specific alcohol consumption—males: moderate = 1–20 g/day, increased = ≥20 g/day; females: moderate = 1–10 g/day, increased = ≥10 g/day

^b^Sum of the following life style factors: smoking, physical activity, alcohol consumption, BMI

We found regional variations concerning lifestyle habits and common chronic diseases. In KORA, participants were less likely to have a history of hypertension, diabetes, stroke or cancer compared to participants in SHIP or in the DHS. In contrast, the 6-month prevalence for any headache (54.55%) and for TTH was higher in KORA compared to the other study regions (Table 1).

In Tables 2, 3, 4, and 5, the association between lifestyle factors and headache subtypes is summarized. With regard to BMI and smoking status, we found inconsistent results for all headache types across the three studies and no clear pattern could be observed for the association between these two life style factors and headache prevalence (Tables 2, 3, 4, 5). Participants who exercised ≥2 h/week had a slightly decreased, but statistically non-significant risk of having complete and probable TTH in every study (Tables 4, 5). For migraine headache, we found inconsistent results across all three studies for physical activity as exposure variable (Tables 2, 3).

Increased alcohol consumption tended to be associated with a decreased risk of experiencing migraine and probable TTH, but without statistically significant results in all of the three studies (Tables 2, 3, 5). With regard to complete TTH, no clear pattern could be observed concerning the relationship with alcohol consumption (Table 4).

The association between the health index and headache is summarized in tables Tables 2, 3, 4, 5. The health index is a sum of risk factors representing an individual risk profile. There was no explicit association between the health index and headache subtypes in any of the three studies.

## Discussion

In this cross-sectional analysis of data from three different studies in Germany, we found two noteworthy results. First of all, we found considerable variations in the distribution of lifestyle factors between the three regions. Second, there was no consistent association between migraine or TTH and the lifestyle factors smoking, alcohol consumption, physical activity and BMI irrespective of the prevalence of a specific lifestyle factor in one of the regions. In addition, a health index reflecting a sum score of four lifestyle factors was not associated with headache subtypes.

Several studies have evaluated a possible association between single lifestyle factors and headache with mostly conflicting results. A negative influence of alcohol consumption on headache occurrence, in general, and headache subtypes have been discussed for years with inconsistent findings. Several cross-sectional studies found no association between alcohol consumption and headache prevalence [10, 13] as well as alcohol consumption and headache subtypes [17]. In contrast, two large population-based studies reported a relationship, however, in opposing directions. In the first study, Aamodt et al. found a significant trend of decreasing prevalence of migraine with increasing amounts of alcohol consumption among 51,383 participants of the Head-HUNT Study. For non-migrainous headache, the inverse association between alcohol consumption and headache prevalence was weaker [8]. In the second study, women but not men who sometimes to often consumed large amounts of alcohol reported more often recurrent headache and migraine [14]. In contrast to our study, headache was not diagnosed according to the IHS criteria in this study. Thus, misclassification is possible and the comparability to other results is limited.

Inconsistency in published results also exists with regard to a possible relation between smoking and headache subtypes [8, 14, 17, 27–31]. In a longitudinal study, Waldie et al. found that the risk of frequent headache was increased among smokers compared to non-smokers in childhood and adolescents. But during adulthood, smoking was not associated with frequent headache. The frequent headache group included migraine cases, TTH sufferers and those with coexisting migraine and TTH. In agreement with Waldie et al., we found no association between smoking status and headache subtypes in our adult population. Two additional cross-sectional studies support our findings [17, 29]. In contrast, the prevalence of both, migraine and non-migrainous headache, increased with higher smoking levels in the Head-HUNT study in participants under the age of 40 years. No association was observed in higher age groups [8]. Our results which were based on the older age range of 35–75 years are in line with the latter finding.

The association between physical activity and headache has gained popularity since case reports and small clinical-based studies suggested that physical activity may reduce headache severity, frequency and activity [11, 12, 15, 32, 33]. Subsequently, several large-scale population studies have been conducted to evaluate the potential association between physical activity and headache prevalence, with mostly conflicting results [14, 16, 18, 19, 29, 34, 35]. In a prospective study, low physical activity was associated with higher prevalences of migraine and non-migraine headache at baseline. After 11 years of follow-up, physically inactive persons were more likely to have non-migraine headache than active individuals [18]. In contrary to these results and consistent with another cross-sectional study [29], we found no significant association between physical activity and migraine prevalence.

Some studies suggested an association between obesity, a consequence of lifestyle, and headache [36–38]. With regard to migraine status, results from several cross-sectional studies indicated that BMI and obesity are not related to migraine prevalence [9, 39, 40]. In agreement with these results, we found no association between migraine fulfilling complete IHS criteria and BMI or probable migraine and BMI. There is increasing evidence that obesity is associated with migraine frequency and, therefore, a risk factor for chronic migraine. Only few studies have evaluated the association between BMI and TTH to date. In one of these studies conducted in 27 states in Brazil, Queiroz et al. found no significant association between TTH and BMI [16].

A possible association between lifestyle factors and headache would have considerable consequences for the prevention of headache. By modifying their individual lifestyle habits, headache sufferers would have the opportunity to influence the occurrence, frequency and severity of headache. As presented before, the results of studies investigating the association between lifestyle factors and headache are not consistent. Methodological differences in the assessment of lifestyle factors and the headache case definition may be one cause for the observed inconsistency. The majority of studies including our own are cross-sectional studies. No assumptions regarding a causal relationship or a time sequence between lifestyle variables and headache are possible in this design. Thus, if, e.g., some headache patients report specific life style behaviours, especially alcohol consumption, to be trigger factors for their headache, they avoid the exposure. Also, patients with severe and frequent headache might not have the ability to exercise on a regular basis [41]. A prospective study design with incident case assessment can better contribute to the clarification of a causal relationship. Nevertheless, our study suggests considerable regional variations with regard to life style habits within one country. The associations between the four lifestyle factors and headache types were negative in all three regions included, regardless of the absolute level of a specific behaviour. We interpret this as a support for a true negative finding.

Our study has several strengths including the large sample size, inclusion of participants from three different regions within one country and a standardized headache assessment by face-to-face interviews. In addition, we classified migraine and TTH and their probable forms using the diagnostic criteria of the 2004 edition of the IHS. Furthermore, information on a variety of lifestyle factors and cardiovascular disease risk factors was available. Several limitations should also be considered when interpreting our results. First, the information on lifestyle variables and headache symptoms is self-reported and we can not rule out potential underreporting, e.g. in alcohol consumption and smoking. Second, assessment of 2 lifestyle variables, physical activity and alcohol consumption, varied slightly between the three studies and we had to transform the information into uniform categories. However, evaluating the association with the original variables in each study yielded no different results (data not presented). Third, we had to exclude over 200 participants in SHIP due to missing data on the exposure status, mainly alcohol consumption. Therefore, we cannot rule out potential selection bias for this part of the analysis. Fourth, among headache sufferers, a higher percentage of participants in SHIP have been diagnosed with probable migraine and probable TTH compared to the DHS and KORA. Since the data collection and headache classification was completely standardized with trained interviewers across all studies, this is unlikely to be due to selection or recall bias. A potential explanation might be a difference in the perception of headache, including the questions from the headache module, in the north-eastern population compared to the two other studies. Fifth, because of the cross-sectional design of our study, a statement concerning a causal relationship or the time sequence of an association cannot be done. Sixth, the cohorts consisted of participants aged 35–75 years from three different regions in Germany which may limit the generalizability of our results to other populations. Lastly, headache is highly prevalent among subjects aged 18–35 years. Since we could not include subjects in this age range, our presented headache prevalence is lower compared to other studies assessing headache prevalence. However, we do not believe that the association between headache and lifestyle depends on headache prevalence and our population is suitable for studying the relationship between lifestyle factors and headache.

In summary, in this large study, we found variations in lifestyle factors in three regions in Germany, but no associations between single or combined life style factors and headache types were observed. Future research should focus on the association of lifestyle habits with first or recurrent headaches.

## Electronic supplementary material

Below is the link to the electronic supplementary material.

Supplementary material 1 (DOC 42 kb)

## Abbreviations

TTH: Tension-type headache
BMI: Body mass index
IHS: International Headache Society
DMKG: German Migraine and Headache Society
SHIP: Study of Health in Pomerania
KORA: Cooperative Health Research in the Region of Augsburg
MONICA: Monitoring Trends and Determinants in Cardiovascular Disease
DHS: Dortmund Health Study
