# Supplementary material for: Association between lifestyle factors and headache
Source: J Headache Pain. 2011 Jan 11;12(2):147–55. doi: 10.1007/s10194-010-0286-0 (PMC3072498; doi:10.1007/s10194-010-0286-0)
Supplement: Supplementary file 1 — Supplementary material 1 (DOC 42 kb) [file 10194_2010_286_MOESM1_ESM.doc]

**Lifestyle questions**

The questions were translated by the authors from the original German questionnaires. Please note that this is not an official translation. The sole purpose of this translation is to provide the reader an overview of the used lifestyle questions in the three cohorts.

SHIP

**Alcohol consumption**

During the last 30 days (4 weeks, 1 month), on how many days have you consumed beer, wine/sparkling wine or spirits (e.g. schnapps, cognac, whiskey, liqueur, cocktails)?

During the last months, on how many days have you consumed beer?

During the last months, on how many days have you consumed wine/sparkling wine?

During the last month, on how many days have you consumed spirits?

Referring to the last 30 days (4 weeks, 1 month), how many glasses of beer, wine/sparkling wine or spirits have you consumed on average per day (referring to a day you have consumed alcoholic beverages)?

Spirits: small glasses (0.02 l)

Spirits: big glasses (0.04 l)

Beer: small glasses/cans/bottles (0.2-0.33 l)

Beer: big glasses/cans/bottles (0.4-0.5 l)

Wine/sparkling wine: glasses (0.2-0.25 l)

**Smoking**

Do you currently smoke or have you ever smoked on a daily basis in the past?

**Physical activity**

Do you exercise/work out?

Which kind of exercise do you perform mostly?

How many hours per week are you spending on this exercise?

Do you perform a second kind of exercise?

How many hours per week are you spending on the second kind of exercise?

DHS

**Alcohol consumption**

How much beer, wine or liquor did you drink over the previous weekend (Saturday and Sunday)?

Beer (0.5 l)

Wine or champagne (0.2 l)

Liquor (number of glasses a 0.02 l)

How much beer, wine or liquor did you drink during the previous workday?

Beer (0.5 l)

Wine or champagne (0.2 l)

Liquor (number of glasses a 0.02 l)

**Smoking**

Do you currently smoke cigarettes, pipe or cigars?

No, never smoked

No, but smoked in the past

Yes

**Physical activity**

How often do you exercise (e.g. running, walking, playing soccer, bicycling, swimming etc)?

Regularly > 2 hours/week

Regularly 1-2 hours/week

Less then 1 hour per week

No exercise

KORA

**Alcohol consumption**

How much beer, wine or liquor did you drink over the previous weekend (Saturday and Sunday)?

Beer (0.5 l)

Light beer (0.5 l)

Alcohol-free beer (0.5 l)

Wine or champagne (0.2 l)

Liquor (number of glasses a 0.02 l)

How much beer, wine or liquor did you drink during the previous workday?

Beer (0.5 l)

Light beer (0.5 l)

Alcohol-free beer (0.5 l)

Wine or champagne (0.2 l)

Liquor (number of glasses a 0.02 l)

**Smoking**

Do you currently smoke cigarettes?

Did you ever smoke cigarettes?

**Physical activity**

How often do you carry out sports in the winter?

Regularly more than 2 hours/week

Regularly 1 to 2 hours/week

Less than 1 hour/week

No sporty activity in winter

How often do you carry out sports in the summer?

Regularly more than 2 hours/week

Regularly 1 to 2 hours/week

Less than 1 hour/week

No sporty activity in summer
